# A Case of Primary Sclerosing Cholangitis Complicated With Liver Abscess Caused by Hyperviscous Klebsiella pneumoniae

**DOI:** 10.7759/cureus.51277

**Published:** 2023-12-29

**Authors:** Koji Takahashi, Hiroshi Ohyama, Izumi Ohno, Yuichi Takiguchi, Naoya Kato

**Affiliations:** 1 Gastroenterology, Chiba University, Chiba, JPN; 2 Medical Oncology, Chiba University, Chiba, JPN

**Keywords:** klebsiella pneumoniae (kp), primary sclerosing cholangitis, pancreaticoduodenectomy, liver abscess, hyperviscous

## Abstract

Liver abscesses caused by *Klebsiella pneumoniae *with a positive string test for hyperviscosity are more likely to develop invasive conditions than those with a negative string test. Here, we report the case of primary sclerosing cholangitis (PSC) who developed a treatment-resistant liver abscess caused by hyperviscous *Klebsiella pneumoniae*. A 67-year-old woman with PSC and a history of pancreaticoduodenectomy developed a fever. She had recurrent bacterial cholangitis after pancreaticoduodenectomy. This time, she was diagnosed with a liver abscess and bacterial cholangitis and then admitted to a local hospital. As her condition did not improve with intravenous administration of meropenem, she was transferred from another hospital to our hospital on the 7th day of admission. The percutaneous transhepatic abscess drainage was performed, and intravenous administration of cefepime and metronidazole was started. *Klebsiella pneumoniae* with a positive string test was detected in the blood culture test and the pus culture of the liver abscess. Although the liver abscess was reduced in size, the infection did not subside completely. Her general condition gradually deteriorated. She passed away on the 45th day of illness. In PSC patients, the formation of a liver abscess caused by hyperviscous *Klebsiella pneumoniae* can be life-threatening. In such cases, pus should be collected as soon as possible to identify the causative bacteria.

## Introduction

Liver abscesses can be caused by *Entamoeba histolytica* or bacteria. When the cause is bacteria, the route to the liver is classified as via the bile duct or the portal vein. *Klebsiella pneumoniae* is the causative agent responsible for approximately 70% of bacterial liver abscesses [1,2]. Liver abscess caused by *Klebsiella pneumoniae* often develops owing to underlying diseases, such as diabetes, liver disease, biliary tract disease, small intestine disease, colon disease, or malignant tumor [3]. Cases of *Klebsiella pneumoniae* with a positive string test for hyperviscosity are more likely to develop invasive conditions than those with a negative string test. This hyperviscous strain of *Klebsiella pneumoniae* is known to cause infection even in immunocompetent individuals and can lead to serious conditions. Here, we report the case of a primary sclerosing cholangitis (PSC) patient who developed a treatment-resistant liver abscess caused by hyperviscous *Klebsiella pneumoniae*.

## Case presentation

A 67-year-old woman with PSC developed a fever and visited a local hospital. She was admitted to the hospital with a diagnosis of liver abscess and bacterial cholangitis. Immediately, treatment was started with 0.5 g of meropenem administered intravenously every eight hours, but there was little improvement in her condition. Her blood pressure dropped, requiring the intravenous administration of noradrenaline. Moreover, pleural effusion developed, and oxygen administration was required. She was transferred to our hospital on the 7th day of her illness for Intensive care management.

**Figure 1:**
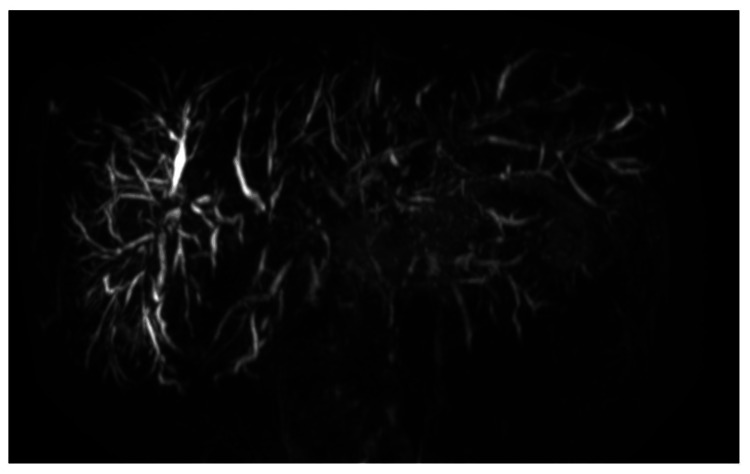
Magnetic resonance cholangiopancreatography after pancreaticoduodenectomy The extrahepatic bile duct was removed by pancreaticoduodenectomy. There were multiple strictures in the intrahepatic bile ducts.

She had chronic heart failure and PSC as a comorbidity. She was diagnosed with PSC 11 years ago and developed jaundice later. At that time, the extrahepatic bile duct stricture was severe, and the guidewire could not pass the biliary stricture during endoscopic retrograde cholangiopancreatography. She was unable to undergo biliary dilatation or drainage. Therefore, she underwent pancreaticoduodenectomy. She had recurrent episodes of bacterial cholangitis after pancreaticoduodenectomy, which always improved with antibiotic treatment until then. There were also multiple strictures in the intrahepatic bile duct (Figure 1). She had no other comorbidities or diseases that could cause immunodeficiency, and she was not taking any immunosuppressants.

At the time of transfer to our hospital, her vital signs were as follows: body temperature, 37.4 °C; respiratory rate, 22 breaths/min; consciousness level, E3V5M6 in Glasgow Coma Scale; blood pressure, 96/62 mmHg (under norepinephrine administration); pulse rate, 64 /min; saturation of percutaneous oxygen, 99% (under oxygen 3 L/min administration). Blood tests showed elevated hepatobiliary enzymes and a severe inflammatory response, as well as findings of prolonged coagulation time and increased fibrin degradation product (Table 1). 

**Table 1:** Laboratory data at the time of transfer to our hospital WBC, white blood cell; RBC, red blood cell; hemoglobin; Hct, hematocrit; MCV, mean corpuscular volume; MCH, mean corpuscular hemoglobin; MCHC, mean corpuscular hemoglobin concentration; Plt, Platelet; TP, total protein; Alb, albumin; BUN, blood urea nitrogen; Cre, creatinine; Na, sodium; K, potassium; AST, aspartate aminotransferase; ALT, alanine transaminase; LDH, lactate dehydrogenase; ALP, alkaline phosphatase; γ-GTP, gamma-glutamyl transpeptidase; T.Bil, total bilirubin; D.Bil, direct bilirubin; Amy, amylase; CK, creatine kinase; CRP, C reactive protein; PCT, procalcitonin; PT, prothrombin; APTT, activated partial thromboplastin time; FDP, fibrin degradation product; BNP, B-type natriuretic peptide

Parameter	Value	Reference Range
WBC	23,700 /μL	3,300-8,600
RBC	363×10^4 ^/μL	386-492
Hb	11.5 g/dL	11.6-14.8
Hct	33.5 %	35.1-44.4
MCV	92.3 fl	83.6-98.2
MCH	31.7 pg	27.5-33.2
MCHC	34.3 g/dL	31.7-35.3
Plt	4.9 ×10^4 ^/μL	15.8-34.8
TP	4.6 g/dL	6.6-8.1
Alb	1.6 g/dL	4.1-501
BUN	45 mg/dL	8-20
Cre	0.82 mg/dL	0.46-0.79
Na	132 mEq/L	138-145
K	3.7 mEq/L	3.6-4.8
AST	547 IU/L	13-30
ALT	304 IU/L	7-23
LDH	471 IU/L	124-222
ALP-IF	106 IU/L	38-113
γ-GTP	117 IU/L	9-32
T.Bil	2.6 mg/dL	0.4-1.5
D.Bil	1.9 mg/dL	0.0-0.2
Amy	93 mg/dL	44-132
CK	429 IU/L	41-153
CRP	29.6 mg/dL	0.00-0.14
PCT	132.0 mg/dL	<0.25
PT activity	57 %	73-118
APTT second	25.3 sec	24.3-34.6
FDP	60.3 μg/mL	<3.9
BNP	73.1 pg/mL	<18.4

**Figure 2:**
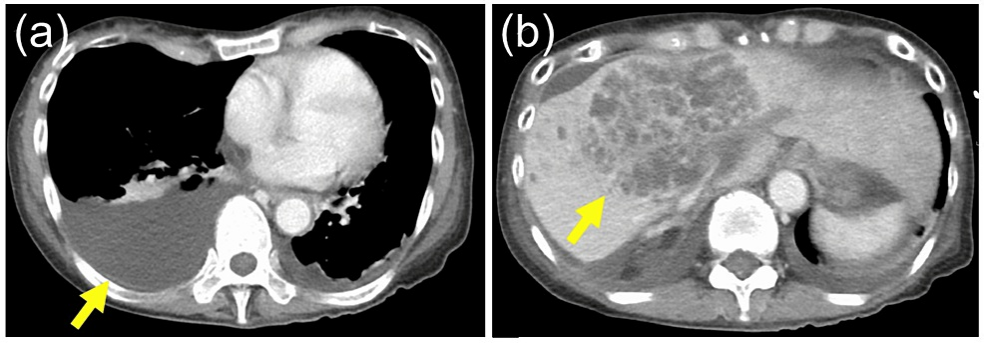
Contrast-enhanced computed tomography images at the time of transfer to our hospital Contrast-enhanced computed tomography at the time of transfer to our hospital revealed pleural effusion (a), an abscess of 10 cm in diameter in the liver anterior segment (b).

Contrast-enhanced computed tomography (CT) scan at the time of transfer to our hospital showed pleural effusion, an abscess of 10 cm in diameter in the liver anterior segment, and ascites (Figure 2). At the time of her transfer to our hospital, she was suffering from septic shock due to a liver abscess and acute cholangitis, as well as disseminated intravascular coagulation due to bacterial infection. Intravenous administration of meropenem did not improve the patient's condition, and we judged that further infection control measures were needed immediately.

**Figure 3:**
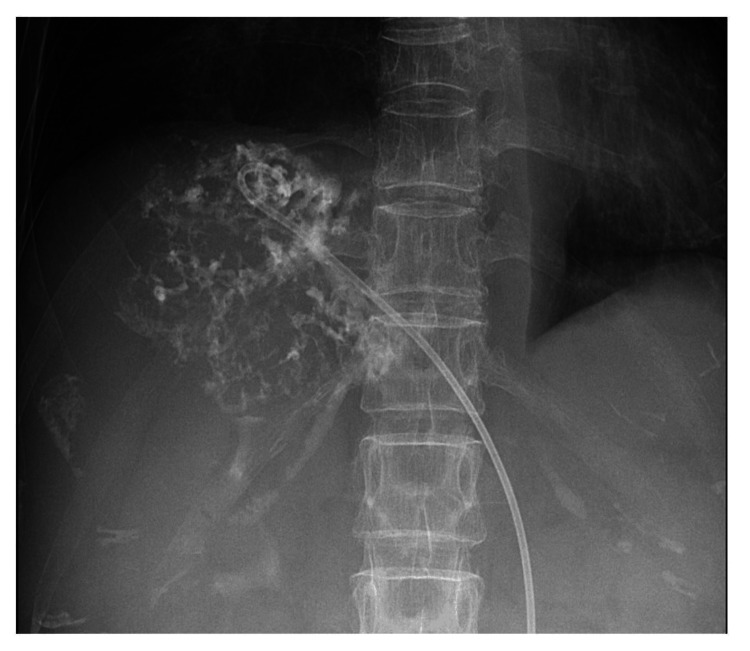
Fluoroscopic image of percutaneous transhepatic abscess drainage For infection control, percutaneous transhepatic abscess drainage was performed on the day of transfer to our hospital. When contrasting the liver abscess cavity, there were numerous septa within the liver abscess with small quantities of fluid.

**Figure 4:**
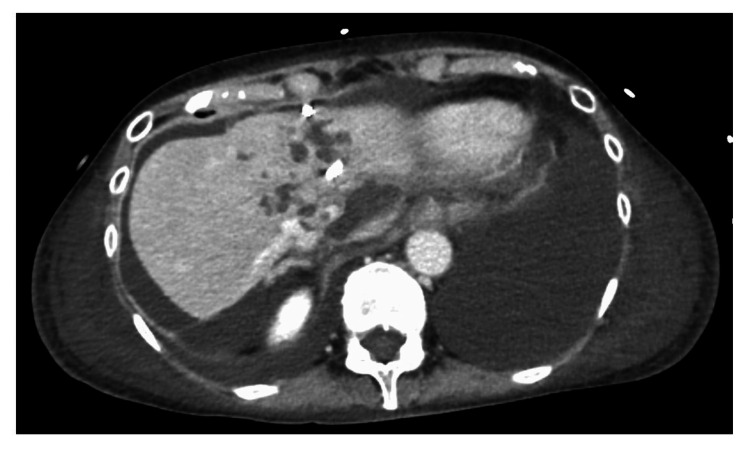
Contrast-enhanced computed tomography images on the 25th day of her illness Contrast-enhanced computed tomography images on the 25th day of her illness revealed that the liver abscess was reduced in size, but the pleural effusion and ascites were increasing.

Percutaneous transhepatic abscess drainage (PTAD) was performed on the day of transfer to our hospital (Figure 3). When contrasting the liver abscess cavity, there were numerous septa within the liver abscess with small quantities of fluid. After consulting with an infectious disease physician, the antibiotics administered after transfer to our hospital were intravenous administration of cefepime 2 g every 12 hours and metronidazole 500 mg every 8 hours. *Klebsiella pneumoniae* with a positive string test was detected in the blood culture test and the pus culture of the liver abscess. Subsequent drug susceptibility tests revealed that *Klebsiella pneumoniae* was sensitive to many antibiotics, including penicillins and cephems. An ophthalmological examination revealed no endophthalmitis. The PTAD tube was frequently occluded, so it was exchanged repeatedly and intravenous antibiotics administration continued. A contrast-enhanced CT scan on the 25th day of her illness showed that the size of the liver abscess had reduced; however, the pleural effusion and ascites had increased (Figure 4). Additionally, her blood pressure improved, and she no longer needed norepinephrine.

Although the liver abscess was reduced in size, the patient had repeated febrile episodes over a long duration and the infection did not subside. The patient was evaluated using a contrast-enhanced CT scan of the whole body, abdominal echo, and cardiac ultrasound examination, but no other sources of infection were found other than acute cholangitis and liver abscess. Her pleural fluid and ascitic fluid were collected by puncture, but there was no evidence of bacterial infection. Then, the patient began to experience frequent acute exacerbations of chronic heart failure, and her general condition gradually deteriorated. Finally, she passed away on the 45th day of her illness.

## Discussion

We believe that there are three reasons why this case's condition became serious. First, *Klebsiella pneumoniae* was hyperviscous; second, it was post-pancreaticoduodenectomy; and finally, PSC coexisted. The combination of these things makes this case difficult to treat and is also what makes this case unique.

*Klebsiella pneumoniae* is a common nosocomial and a potential community‑acquired pathogen, which can cause infections in all age groups, especially in the immunocompromised. In 1986, a case of liver abscess caused by hypervirulent *Klebsiella pneumoniae* complicated by sepsis owing to endophthalmitis was reported in Taiwan [4]. These hypervirulent strains are capable of creating foci of infection in various organs in the body, some of which may be life-threatening [5]. Moreover, the emergence of hypervirulent strains broadens the occurrence of infection among healthy and immunocompetent individuals [6,7]. Hypervirulent strains are characterized by high colony viscosity. The test for hyperviscosity is called the “string test”, and it is considered positive if the viscous string extends by 5 mm or more from the colony on the blood agar plate. Among the capsular serotypes identified to date, K1 and K2 serotypes are mostly reported in invasive diseases and are known to be hypervirulent [8]. In this case, the strain of *Klebsiella pneumoniae* was string test positive, it is unclear whether the strain was a hypervirulent, because strain genetic testing had not been performed. 

There are several reports regarding the relationship between pancreaticoduodenectomy and liver abscess. In 2019, Chen et al. analyzed the data of 326 patients who underwent pancreaticoduodenectomy and reported that diabetes was a risk factor for developing liver abscess after pancreaticoduodenectomy. In that report, multiple liver abscesses were more common than single ones, and *Klebsiella pneumoniae* was the most common causative agent of liver abscesses [9]. In 2014, Njoku et al. analyzed the data of 1,189 patients who underwent pancreaticoduodenectomy or distal pancreatectomy and reported that the risk factors for developing liver abscess after pancreaticoduodenectomy were postoperative biliary fistula and need for reoperation [10]. Diabetes was not a comorbidity in this case. Furthermore, no biliary fistula or reoperation occurred after pancreaticoduodenectomy. It is unclear whether PSC is a factor that increases the likelihood of developing liver abscess after pancreaticoduodenectomy, as there are no previous reports about the relationship.

PSC is an idiopathic, progressive, cholestatic liver disease characterized by patchy, non-bacterial inflammation, fibrosis, and stricture. The pathogenesis of PSC remains unknown [11,12]. After diagnosis of PSC, the reported median survival is 12 to 15 years [13,14]. It was reported that PSC is a risk factor for the development of acute bacterial cholangitis, and approximately 30% to 40% of patients with PSC have a history of bacterial cholangitis [15]. In addition, although there is a small number of cases (less than 10% of the total), there are certain cases of PSC with repeated episodes of bacterial cholangitis, but the factors contributing to recurrent bacterial cholangitis are unknown [16]. Although death from bacterial cholangitis itself is rare, it causes complications such as liver abscess, osteomyelitis, and endocarditis. These complications greatly affect the overall morbidity and mortality of PSC patients [16]. 

This patient had recurrent bacterial cholangitis after pancreaticoduodenectomy. Although this case is a unique condition, having undergone pancreaticoduodenectomy, it is classified into the group of recurrent bacterial cholangitis among PCS. A plausible reason for her repeated episodes of cholangitis is that bacteria from the jejunum can easily enter the bile ducts through choledochojejunostomy. Additionally, owing to PCS, the patient’s intrahepatic bile duct had multiple strictures, making it difficult to remove bacteria that had entered the bile duct. In this case, we assumed that *Klebsiella pneumoniae* invaded the bile duct and caused acute cholangitis, which led to the development of a liver abscess. In this case, various factors combined, including hyperviscous *Klebsiella pneumoniae* as the causative agent, post-pancreaticoduodenectomy, and PSC as a comorbidity, resulted in a severe condition that led to serious conditions. The condition could have been improved by draining the abscess earlier. It is noteworthy that the formation of a liver abscess caused by hyperviscous *Klebsiella pneumoniae* can be life-threatening in PSC patients. In such cases, pus should be collected as soon as possible to identify the causative bacteria.

## Conclusions

We reported the case of a PSC patient who developed a treatment-resistant liver abscess caused by hyperviscous *Klebsiella pneumoniae*. In this case, a combination of various factors contributed to the severity of the condition. In PSC patients, liver abscess due to hyperviscous *Klebsiella pneumonia* can be life-threatening. Early identification of the causative agent is necessary for liver abscesses formed in PSC patients with repeated bacterial cholangitis.
